# Cross-Reactivity between Half Doses of Pfizer and AstraZeneca Vaccines—A Preliminary Study

**DOI:** 10.3390/vaccines10040521

**Published:** 2022-03-27

**Authors:** Krzysztof Lukaszuk, Amira Podolak, Paulina Malinowska, Jakub Lukaszuk, Grzegorz Jakiel

**Affiliations:** 1 Invicta Research and Development Center, Polna 64, 81-740 Sopot, Poland; luka@gumed.edu.pl (K.L.); jakub.lukaszuk@invicta.pl (J.L.); grzegorz.jakiel1@o2.pl (G.J.); 2 Department of Obstetrics and Gynecological Nursing, Faculty of Health Sciences, Medical University of Gdansk, 80-210 Gdansk, Poland; 3 1st Department of Obstetrics and Gynecology, The Center of Postgraduate Medical Education, Zelazna 90, 01-004 Warszawa, Poland

**Keywords:** COVID-19, vaccines, mixed doses, immune response, Pfizer, AstraZeneca, vaccine equity

## Abstract

Media reports have caused a significant drop in confidence in the AstraZeneca ChAdOx1 nCoV-19 COVID-19 vector vaccine (Vaxzevria, AstraZeneca Södertälje, Sweden). This has caused many people, already vaccinated with the first dose of AstraZeneca, to refuse vaccination with this product. On the other hand, the increased demand for mRNA vaccines has resulted in a greater shortage of mRNA vaccines and cases of people being vaccinated with the AstraZeneca vaccine after the first dose of the Pfizer/BioNTech BNT162b2 COVID-19 vaccine (Comirnaty, Pfizer/BioNTech, Mainz, Germany). Moreover, currently, 60.9% of the global population have received at least one dose of a COVID-19 vaccine, while only 10% of people in low-income countries have received at least one dose. Even less people are fully vaccinated. The present pilot study evaluated the administration of half doses of AstraZeneca and Pfizer vaccines and included the enrollment of 26 subjects who were vaccinated with a different vaccine the first and second time. The reference group included individuals undergoing vaccination with two full doses of the Pfizer vaccine (21-day interval) monitored for their antibody levels as part of a parallel study. The distribution of antibody levels was not significantly different between those who received the Pfizer vaccine alone and those receiving the AstraZeneca vaccine plus Pfizer or Pfizer and AstraZeneca. To prepare for the next pandemic waves, solving the problem of the matching of booster vaccine to the previously received doses would be advisable. The topic is important and emerging as most of the population in low-income countries is still not vaccinated. We strongly believe that vaccine equity is the most important aspect of vaccination strategies.

## 1. Introduction

Coronavirus disease-19 (COVID-19) is the disease caused by SARS-CoV-2 virus, which was reported for the first time in December 2019 in Wuhan, China. Since then, it has spread all over the world. The symptoms of the diseases originally included fever, tiredness, dry cough, and, later, also the loss of taste and smell. However, the symptoms ceased to be so characteristic as the new variants spread [1].

The COVID-19 pandemic has induced the advancement in technologies for virus detection. SARS-CoV-2 RNA can be easily detected with the use of quantitative reverse transcriptase–polymerase chain reaction [1]. Additionally, there are also several commercially available SARS-CoV-2 antibody tests [2]. Moreover, next-generation sequencing plays a crucial role in evaluating new variants’ transmittance, virulence, as well as resistance to the available vaccines [1]. Furthermore, numerous researches have been performed to evaluate the prognostic markers of SARS-CoV-2 infection [3,4,5,6]. For instance, S100 family genes in nasal swabs turned out to be predictive for disease severity [3].

The COVID-19 pandemic has also started the turn in vaccine research [7]. Currently, there are several COVID-19 vaccines authorized for use by the European Medicines Agency (EMA) and U.S. Food and Drug Administration (FDA) (Table 1). The approved vaccines are based on different technologies, including mRNA and vector vaccines. For instance, the Pfizer/BioNTech BNT162b2 COVID-19 vaccine contains mRNA with instructions for making the spike protein—the protein on the virus’ surface, needed to enter the body’s cells. The AstraZeneca ChAdOx1 nCoV-19 vaccine is made up of adenovirus which was modified to contain the gene encoding above-mentioned protein [8,9]. Following vaccination, with either type of a vaccine, cells of a human body temporarily produce the spike protein. In consequence, the immune system recognizes the protein, produces antibodies, and activates T cells. Neither mRNA, nor the adenovirus vector stay in the body after vaccination. For now, only the BNT162b2 vaccine is authorized for use in children (above 5 years old). Similarly, the BNT162b2 vaccine is also recommended for use during pregnancy, as a large amount of data indicated no increase in pregnancy complications during the second and third trimester. There are very limited data on the use of ChAdOx1 during pregnancy.

Media reports have caused a significant drop in confidence in the AstraZeneca ChAdOx1 nCoV-19 (AZ) COVID-19 vector vaccine (Vaxzevria, AstraZeneca, Södertälje, Sweden) [12]. According to current recommendations, Vaxzevria must not be given to people who have had thrombosis with thrombocytopenia syndrome after receiving the vaccine. Additionally, it must also not be given to people who have previously had capillary leak syndrome [9]. This has caused many people, already vaccinated with the first dose of AstraZeneca, to refuse vaccination with this product. On the other hand, the increased demand for mRNA vaccines has resulted in a greater shortage of mRNA vaccines and cases of people being vaccinated with the AstraZeneca vaccine after the first dose of the Pfizer/BioNTech BNT162b2 COVID-19 (Pf) vaccine (Comirnaty, Pfizer/BioNTech, Mainz, Germany). Furthermore, the main problem in vaccine strategy seems to be low vaccine supply in low- and middle-income countries. Even now, when 24.96 million doses are administered each day, only 10% of people in low-income countries have received at least one dose. For instance, in Ethiopia, 7.95% of the population are vaccinated with at least one dose, whereas only 1.36% are fully vaccinated. The situation in Madagascar is even worse; only 2.88% of the population are fully (two doses) or partially vaccinated (one dose) [13]. The global vaccine inequity is taking its toll on some of the world’s poorest and most vulnerable people. Additionally, it means that the virus will continue to mutate and, in consequence, widespread havoc around the globe [14].

Apart from vaccine undersupply, the costs of mRNA vaccines also need to be considered [15]. Lowering vaccination costs for low- and middle-income countries is only a step towards enabling them to benefit from universal vaccination. An entire infrastructure must be created to ensure rapid, inexpensive, and safe population vaccination in countries lacking advanced technological and administrative infrastructure. One of the elements that can be considered is a dose-reduction strategy. Such a solution was effective against yellow fever in Africa and South America [16]. Moreover, we have already demonstrated no statistically significant differences between the mean antibody levels in the serum of the subjects under 55 years of age vaccinated with the recommended dose and half dose of BNT162b2 [17]. Similarly, a quarter dose of mRNA-1273 was found to provide comparable levels of both antibodies and T cells to those induced by natural infection [18]. For now, the main goal to achieve is to test the possibility of using vaccines interchangeably. If confirmed, a reduction of the administrative requirements for successive vaccinations with vaccines from the same manufacturer should be considered. It is understandable that vaccine manufacturers conduct clinical trials and promote the use of their own vaccines. However, their point of view may result more from commercial business needs than purely scientific and clinical needs. Therefore, studies independent from vaccine manufacturers are needed. The ongoing Com-COV study is evaluating the administration of full doses of AZ and Pf vaccines in a heterogenous prime-boost schedule [19]. We have considered our previous results from the study assessing the homogenous prime-boost half-dose schedule, and set out to evaluate the humoral response of patients receiving half doses of AZ and Pf vaccines in a heterogenous prime-boost schedule [17].

## 2. Materials and Methods

### 2.1. Ethical Policy

The study evaluated the administration of half doses of AZ and Pf vaccines and included the enrollment of 26 subjects who were vaccinated with a different vaccine the first and second time. The ethics committee approvals were obtained for this study (Ethics Committee Gdansk Regional Medical Board opinion KB-14/21). All participants gave written informed consent for providing blood samples.

### 2.2. Participants, Vaccination, and SARS-CoV-2 Antibodies Measurement

The inclusion criteria were a willingness to participate in the study and age between 18 and 55 years. Exclusion criteria were the presence of comorbidities or chronic diseases such as diabetes, hypertension, heart disease, chronic pulmonary diseases, and obesity (defined as BMI > 30). The majority of the participants declared interest in the study due to the possibility of earlier vaccination and fear of the side effects after a full dose of the vaccine. The reference group included individuals undergoing vaccination with two full doses of Pf vaccine (21-day interval) monitored for their antibody levels as part of a parallel study (Ethics Committee Gdansk Regional Medical Board opinion KB-4/21). This group consisted of 188 healthcare professionals who were vaccinated as part of the national vaccination program. The mean age of the study group was 37.1 ± 8.9, whereas the mean age of the control group was 38.8 ± 10.7.

A total of 12 cases were vaccinated initially with Pf, and 14 initially with AZ. The second vaccine was given between 21 and 28 days after dose-1. Vaccines were administered intramuscularly in the deltoid muscle of the upper arm. Vaccine lots used were as follows: Pf: ER 7812, EW 9127, EY 7015, FA 5715; AZ: ABV 6514, ABW 0891, ABT 3519. Each participant had blood drawn on the day of the dose-2 and 8–10 days after dose-2.

### 2.3. Assay Characteristics

The study used the Elecsys Anti-SARS-CoV-2 S IVD kits (Roche Diagnostics, Switzerland). This automated assay is certified for in vitro diagnostic (IVD) clinical use and is intended for the quantitative determination of IgG and IgM high-affinity antibodies against SARS-CoV-2 spike (S) protein receptor-binding domain (RBD) in human serum and plasma [2]. It requires 12 uL of samples to perform an analysis. It is based on the electro-chemiluminescence immunoassay (ECLIA) method and the double-antigen sandwich assay test principle. Initially, it was standardized against the internal Roche standard for anti-SARS-CoV-2 S; however, from the date 12 January 2021 it is also standardized against the World Health Organization (WHO) IS: NIBSC 20-136. The test results are reported in units per mL (U/mL). According to the manufacturer, it can be converted to binding antibody units per mL (BAU/mL)—a WHO standard—by multiplying the Roche result by 1.029. The limit of detection (LoD) was established at 0.36 BAU/mL, whereas the measuring range was 0.41–257 BAU/mL (to 2570 BAU/mL for 10-fold diluted samples) [2]. 

### 2.4. Statistical Analysis

Statistical analyses were performed using R packages (Tidyverse, Rstudio, Boston, MA, USA) [20]. The Wilcoxon signed-rank test was used to assess the differences in mean antibody levels.

## 3. Results

**Figure 1:**
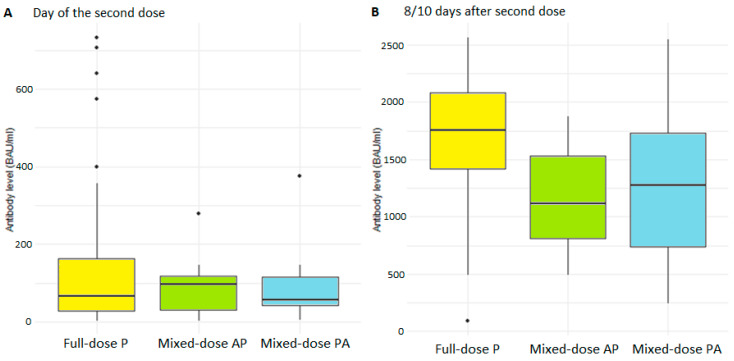
Boxplot (median, hinges: first and third quartiles, whiskers: the largest value no further than 1.5 * IQR from the hinge) showing humoral response to vaccination as measured with antibody levels in the full-dose and mixed-dose groups on the day of the second dose (**A**) and 8–10 days after the second dose for participants whose results did not exceed the test detection limit (**B**).

The distribution of antibody levels was not significantly different between objects who received the Pfizer vaccine alone and those receiving the AZ vaccine plus Pfizer or Pfizer and AZ (Figure 1). They were within the wide variation in humoral response that we observed after Pfizer vaccination. The mean level of antibodies on the day of the second vaccine dose in the reference group was 113 BAU/mL, and 90.5 BAU/mL among subjects who received half doses of different vaccines. A total of 8–10 days following the second vaccination dose, 70% of subjects in the reference group and 50% of the study group had results exceeding the detection limit of the Roche test. The mean levels in the subjects that did not exceed the detection limit were 1700 BAU/mL (*n* = 45) and 1230 BAU/mL (*n* = 13) in the reference and study group, respectively.

## 4. Discussion

Rapid population vaccination appears to be the only available solution leading to a return to normalcy. Currently, 63.8% of the global population have received at least one dose of a COVID-19 vaccine, compared with only 14.1% of people in low-income countries. Even less are fully vaccinated [13]. This drives a conclusion that the main problem of vaccine strategy is the vaccine inequity [21,22]. Currently, the International Rescue Committee (IRC) notes that countries such as Burkina Faso and Niger, already requiring significant humanitarian assistance, saw a 31% and 22% increase in COVID deaths, respectively, whereas only 13% of doses contracted by COVID-19 Vaccines Global Access (COVAX, the program supporting global vaccination) have been delivered [23]. Due to vaccine inequity, the Omicron variant spreads the world and wealthy nations consistently prioritize boosters for themselves. Meanwhile, people of low-income countries are still being ignored [23]. For instance, it was calculated that high-income countries have already administered 69 times more doses than Bangladesh [24]. Moreover, it was estimated that approximately 50 million doses in the rich-country stockpile are on the point of expiring before they can be donated and, due to over-buying, the unused stockpile will keep growing well into next year [25].

With the support of the Strategic Advisory Group of Experts (SAGE) in Immunization and its COVID-19 Working Group, WHO published the statement encouraging research in vaccine reduction [26]. We have already demonstrated no differences in humoral response between the groups vaccinated with half and the recommended dose of the BNT162b2 vaccine in subjects under 55 years of age [17]. For now, the main goal is to test the possibility of using vaccines interchangeably. This could be useful in vaccination strategies especially in low-income countries.

Currently, it is advised to preferentially use subsequent doses in a vaccination series from the same manufacturer where possible [27]. On the other hand, for people aged 18 years and older, mRNA vaccines are recommended as a booster dose, regardless of the type of vaccine received for the first two doses. COV-BOOST, a multicenter, randomized, controlled, phase 2 trial of third-dose booster vaccination against COVID-19, was conducted in the United Kingdom. Participants were initially vaccinated with two doses of Pfizer/BioNTech BNT162b2 COVID-19 vaccine (Comirnaty, Pfizer/BioNTech, Mainz, Germany) or ChAdOx1 nCoV-19 COVID-19 vector vaccine (Vaxzevria, AstraZeneca, Södertälje, Sweden). All tested vaccines showed acceptable reactogenicity and side-effect profiles. However, this study did not evaluate the safety and efficacy of mixed vaccines for the first two doses [28].

Nevertheless, conducted studies demonstrated that immunogenicity can be sustained with vaccines made by different manufacturers used in the same individual to complete a vaccination program for certain diseases [29]. 

The present study compares the humoral response to the half dose of mixed vaccines—Pfizer/BioNTech BNT162b2 COVID-19 vaccine (Comirnaty, Pfizer/BioNTech, Mainz, Germany) and ChAdOx1 nCoV-19 COVID-19 vector vaccine (Vaxzevria, AstraZeneca, Södertälje, Sweden)—and the recommended dose of the BNT162b2 vaccine administered to adults up to 55 years old. The obtained results demonstrated that the distribution of antibody levels was not significantly different between those who received the Pfizer vaccine alone and those receiving the AZ vaccine plus Pfizer or Pfizer and AZ.

These findings indicate the possibility of treating the above-mentioned vaccines interchangeably. We already know that the level of antibodies obtained after vaccination decreases rapidly by approximately 48% from the peak on day 8 after the second dose to day 30 after the second dose of the vaccine [30,31]. The study by Liu et al. tested sera, two and four weeks after the second dose, and showed lower geometric mean plaque reduction neutralization titers against variant viruses, including the B.1.617.2 [32]. Garcia-Beltram et al. also showed that emerging mutations of the virus have reduced sensitivity to the neutralizing effect of the binding antibodies formed after vaccination requiring up to 42 higher antibody levels [32,33].

There was one study performed to evaluate the safety of heterologous prime-boost COVID-19 vaccination using the BNT162b2 COVID-19 vaccine and ChAdOx1 nCoV-19 vaccine in people of age 50 years and older with no or mild-to-moderate, well-controlled comorbidity. The study reported higher rates of feverishness, chills, fatigue, headache, joint pain, malaise, and muscle ache in both heterologous vaccine schedules compared with homologous ones [19]. However, there were no hospitalizations due to solicited symptoms, and most of this increase in reactogenicity was observed in the 48 h after immunization. Additionally, hematology and biochemistry profiles were similar between heterologous and homologous vaccine schedules, and no thrombocytopenia was reported in any group at day seven, post-boost [19].

On the other hand, the spread of COVID-19 variants may be a threat to reaching vaccination success. It was demonstrated that primary immunization with the BNT162b2 COVID-19 vaccine and ChAdOx1 nCoV-19 vaccine provided limited protection against symptomatic disease caused by the Omicron (B.1.1.529) variant. However, the booster dose of BNT162b2 or mRNA-1273, after either BNT162b2 or ChAdOx1 primary course, substantially increased protection. Nevertheless, it was found to wane with time [34,35]. Therefore, booster doses may be the solution to mitigate the reduction in vaccine effectiveness. If so, the reduction of vaccine doses, along with mixing them, may help in reaching this goal, especially in low- and middle-income countries.

SARS-CoV-2 is predicted to not be eradicated but rather become endemic alongside other ‘seasonal’ coronaviruses. Most likely, it is expected to evolve more predictably—becoming more infectious and transmissible. For instance, ‘seasonal’ coronavirus 229E infects humans repeatedly throughout their lives. Although, other respiratory viruses, including 229E, indicates the potential future of SARS-CoV-2, the virus may still evolve in several different directions [36]. Nevertheless, in almost every scenario there remains the need of repeated vaccination, once again, driving the advantages of reducing and mixing the doses.

As this was a pilot study, the main limitation is the sample size. Thus, the number of individuals is too small to use these results immediately in the vaccination schedule. However, it has already indicated such a possibility, and we would like to encourage the researchers and suppliers to test the compatibility of vaccines with each other in order to facilitate the logistics and costs. Currently, even AstraZeneca highlights the potential benefits of vaccine interchangeability, as it may promote flexibility in relation to the dose and frequency needed to provide protection, gives more options for administration, allows manufacturing to proceed more quickly, addresses logistical challenges, and increases their overall availability [37].

Moreover, it must be highlighted that our study was performed before including the booster dose in the vaccination schedule. To our knowledge, there are currently several ongoing trials concerning heterologous vaccine strategy—this one is funded by the National Institute of Allergy and Infectious Diseases—in which adult volunteers who have been fully vaccinated against COVID-19 receive booster doses of different COVID-19 vaccines to determine the safety and immunogenicity of mixed booster regimens. The study completion date is estimated to be 1 December 2022 [38]. Additionally, according to clinicaltrials.gov there are several others: the trial sponsored by Canadian Immunisation Research Network, estimated to complete in April 2023 [39], the International Vaccine Institute trial in Mozambique which is reported to not complete until November 2022 [40], and the trial of the National Taiwan University Hospital which is said to complete in July 2022 [41].

Another limitation of our study is that antibody levels sufficient to neutralize the virus and block its entry into the host cell and multiplication are not yet known. Roche manufacturer’s data shows correlation of the antibody levels to serum neutralization with 92.3% positive agreement (95% CI 63.97–99.81%). WHO introduced the International Standard and Reference Panel for the anti-SARS-CoV-2 antibody, with the goal of standardizing the results obtained with different kits and correlating their level with the effect of neutralizing agents. However, its usefulness has not been confirmed in available studies [2,42].

## 5. Conclusions

The optimal solution to prepare for the next pandemic waves would be the creation of targeted vaccines for emerging virus variants. This, however, may be time consuming. Booster vaccinations may be an easier solution to carry out. Such a solution requires solving the problem of the matching of the booster vaccine to the previously received doses. If we can prove that the second dose of the vaccine does not need to match the first dose, it will give us the possibility of accelerating subsequent vaccinations by making them independent of suppliers and logistically correlating patients with a particular vaccine. To this end, it would be advisable to require that subsequent vaccines be tested for compatibility with each other during the registration process, in order to facilitate the logistics of subsequent population revaccination. The topic is not only important but also emerging as most of the population in low-income countries is still not vaccinated. We strongly believe that vaccine equity is the most important aspect of vaccination strategies.

## Figures and Tables

**Table 1:** Overview of approved COVID-19 vaccines. Legend: X = accepted [10,11].

Vaccine	Technology	EMA Authorization	FDA Authorization
**Comirnaty, Pfizer/BioNTech**	mRNA vaccine	X	X
**Vaxzevria, AstraZeneca**	recombinant vaccine	X	
**Spikevax, Moderna**	mRNA vaccine	X	X
**Nuvaxovid, Novavax**	recombinant, adjuvanted vaccine	X	
**Janssen, Johnson&Johnson**	recombinant vaccine	X	X

## Data Availability

The data presented in this study are available on request from the corresponding author.

## Abbreviations

AZ: ChAdOx1 nCoV-19 COVID-19 vector vaccine (Vaxzevria, AstraZeneca); BAU: binding antibody units; BMI: body mass index; COVID-19: coronavirus disease 2019; ECLIA: electro-chemiluminescence immunoassay; EMA: European Medicines Agency; FDA: U.S. Food and Drug Administration; IQR: interquartile range; IRC: International Rescue Committee; IVD: in vitro diagnostic; LoD: Limit of detection; Pf: Pfizer/BioNTech BNT162b2 COVID-19 vaccine; RBD: receptor-binding domain; S: SARS-CoV-2 spike protein; SAGE: Strategic Advisory Group of Experts in Immunization; SARS-CoV-2: severe acute respiratory syndrome coronavirus 2; and WHO: World Health Organization.
